# Rational allocation of coronavirus disease 2019 (COVID-19) vaccines to healthcare personnel and patients: A role for antimicrobial stewardship programs?

**DOI:** 10.1017/ice.2020.1393

**Published:** 2020-12-16

**Authors:** Priya Nori, Payal K. Patel, Michael P. Stevens

**Affiliations:** 1 Division of Infectious Diseases, Department of Medicine, Montefiore Medical Center, Albert Einstein College of Medicine, Bronx, New York; 2 Infectious Diseases Section, Ann Arbor Veterans’ Affairs Medical Center, Ann Arbor, Michigan; 3 Healthcare Infection Prevention Department, Virginia Commonwealth University Health System, Richmond, Virginia

*To the Editor—*Vaccine allocation planning is not traditionally considered a core activity of antimicrobial stewardship programs (ASPs).^1,2^ However, ASP physicians and pharmacists are well suited to participate in health-system–wide coronavirus disease 2019 (COVID-19) vaccination efforts given their expertise, programmatic infrastructure, institutional leadership, and trust within their organizations. Although ASPs have played a central role in the COVID-19 response pertaining to novel therapeutics,^3,4^ little has been published on ASP involvement in COVID-19 vaccine allocation planning. A PubMed search for “antimicrobial stewardship and severe acute respiratory coronavirus virus 2 (SARS-CoV-2) vaccine” and “antimicrobial stewardship and COVID-19 vaccine” on December 2, 2020, returned no relevant results. Given the rapid expansion of their roles during the pandemic, ASPs may be asked to contribute to health system COVID-19 vaccine allocation planning. ASP contributions to similar institutional efforts predate the pandemic. For instance, ASPs hold important roles in institutional pharmacy and therapeutics committees for formulary addition of new vaccines (eg, new recombinant zoster and meningococcal vaccines). Historically, some ASPs were integrated into 2009 H1N1 influenza pandemic response efforts, participating in vaccine planning and the development of antiviral treatment guidelines.^5^

Since the COVID-19 pandemic began, ASPs have developed significant experience in designing rational allocation systems for novel therapeutics such as remdesivir,^4^ which is now incorporated into ASP preauthorization paradigms throughout the country. ASP pharmacists have particular expertise in preparation, transport, and storage required for specific COVID-19 vaccine products. Extreme temperature requirements pose a major challenge for community hospitals and nursing facilities in rural areas without access to medical-grade deep freezers.^6^ ASPs can develop coordinated vaccine distribution systems from a centralized location within health systems to distant facilities. ASPs frequently interface with health information technology departments to design electronic medical record add-ons for stewardship functions, which can be harnessed to create a streamlined order set for all available COVID-19 vaccines and templates for mandatory documentation and reporting required by state health departments.

ASPs have also become a trusted resource for accurate interpretation and dissemination of published data and society guidelines on SARS-CoV-2 therapeutics, such as remdesivir, convalescent plasma, and monoclonal antibodies. Applying this concept to vaccines, ASPs can work with institutional leaders to review data from COVID-19 vaccine trials and adapt recommendations of the Advisory Committee on Immunization Practices (ACIP) on ethical vaccine allocation to their health systems, with the goals of maximizing benefits and minimizing harms, promoting justice and transparency, and mitigating health inequities.^7^ ASPs can also help health systems quickly adapt to evolving guidance from public health authorities in terms of initial target vaccination groups.^7^ Healthcare workers at greatest risk of exposure as well as those caring for the most vulnerable patients will be prioritized in phase 1 of vaccination.^7^ Partnership with local skilled nursing facilities (SNFs) may be required to bolster their resident and associate vaccination efforts. ASP pharmacists and physicians are well suited to provide expertise pertaining to vaccine education, preparation, storage, and monitoring and reporting side effects.

Although ASPs are assets to vaccine allocation planning, many challenges remain. Due to limited initial vaccine doses, a preauthorization and careful tracking mechanism is ideal; however, ASP preauthorization is primarily limited to antimicrobials used in the inpatient setting, and vaccines are outside the scope of this paradigm. Additionally, employee health records systems can exist separately from patient electronic medical records systems, and close coordination with hospital occupational health programs will be required. Another major hurdle is an “infodemic” of COVID-19 misinformation.^8^ However, as mentioned above, ASPs are a trusted source of information, and they have considerable experience educating both HCWs and patients on appropriate antimicrobial use. This skill will prove useful when addressing hesitancy concerning novel vaccines with only short-term safety data. For instance, ASPs can reinforce Centers for Disease Control and Prevention recommendations regarding the role of COVID-19 vaccination in individuals with past infection and evidence of natural immunity.^9,10^ Patient and staff education are routinely conducted by ASP staff.^11^

Notably, ASP involvement in COVID-19 vaccination efforts will require a significant investment of effort at a time when ASPs are already tasked with other important pandemic-related roles (eg, developing outpatient monoclonal antibody infusion programs).^12^ This effort will naturally come at the expense of other ASP activities such as ensuring appropriate perioperative prophylaxis for elective surgical procedures, which may continue at certain hospitals to financially support institutions. Health systems will need to invest in ASPs in terms of dedicated effort and resources to achieve desired outcomes during the pandemic. To mitigate potential loss of other critical ASP functions, tracking, and reporting of vaccinations should be diverted to other hospital personnel after initial ASP-lead education and allocation planning.

**Fig. 1.**
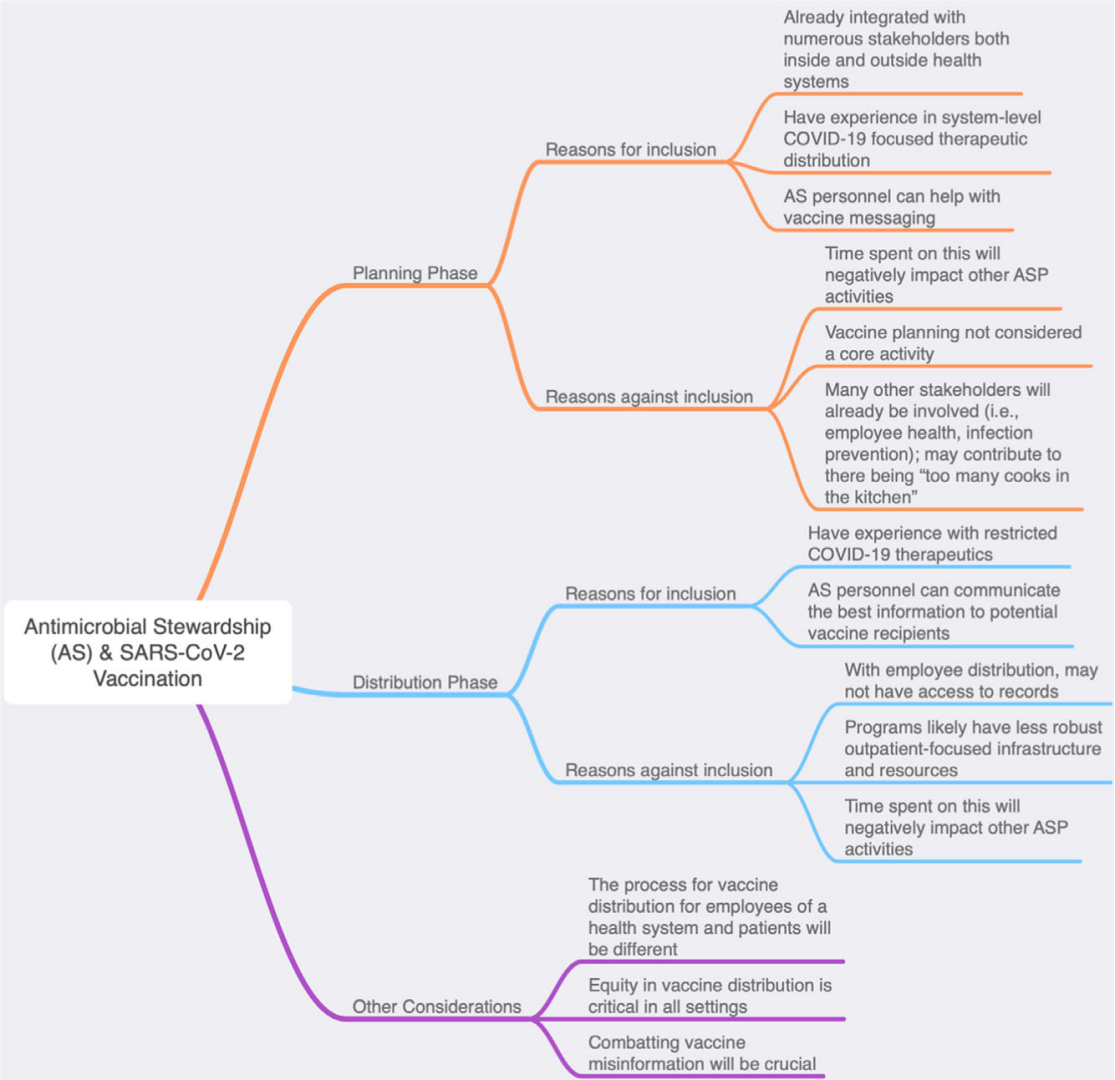
Antimicrobial stewardship (AS) and SARS-CoV-2 vaccination.
